# Digital behavior and anxiety in the post-pandemic era: a five-year analysis of screen time, sleep, and behavioral risk profiles

**DOI:** 10.3389/fpubh.2026.1766808

**Published:** 2026-02-25

**Authors:** Wenjing Liu

**Affiliations:** Department of Business Administration, Sejong University, Seoul, Republic of Korea

**Keywords:** digital behavior, latent profile analysis, post-pandemic mental health, screen time, sleep duration

## Abstract

**Background and aims:**

The post-pandemic period has shown sustained digital intensification associated with adverse mental health outcomes. Focusing on adults, this study examined associations between screen time, sleep duration, social media use, and anxiety in a global sample from 2020 to 2024. It further tested moderation by age and sleep adequacy and identified high-risk behavioral subgroups.

**Methods:**

This study applied hierarchical regression models and person-centered clustering techniques to a publicly available, repeated cross-sectional global dataset on adult mental health and lifestyle (2019–2024; *N* = 3,000). Moderation by age and sleep adequacy was assessed, and behavioral profiles were identified using k-means clustering. Statistical significance was evaluated at *p* < 0.05.

**Results:**

Screen time was positively associated with anxiety, with each additional hour corresponding to a b of 0.30 (*p* < 0.001). Similarly, each additional hour of social media use was associated with a *b* of 0.25 (*p* < 0.001). In contrast, sleep duration showed a protective effect, with each additional hour linked to a b of −0.36 (*p* < 0.001). The association between screen time and anxiety was stronger among adults younger than 40 years (*b* = 0.323) than among those aged 40 years and older (*b* = 0.265), and this age-based interaction was statistically significant (*p* = 0.039). A high-screen and low-sleep behavioral profile, comprising 34.3 percent of the sample, exhibited the highest mean anxiety level (*M* = 6.27).

**Implications:**

Findings support age-stratified interventions emphasizing sleep hygiene and nighttime device boundaries for younger adults. Screening for high-screen and low-sleep behavioral profiles in clinical and educational settings is recommended. Future longitudinal research using objective measures is needed to clarify causality and guide targeted public health strategies.

## 1 Introduction

The post-pandemic period has been marked by a sustained intensification of digital engagement that continues to reshape communication, work, education, and everyday routines (1, 2). Meta-analytic evidence indicates that screen time among adults increased by approximately 1 h per day during the pandemic (3, 4). Current evidence suggests that these elevated levels have persisted rather than returning to pre-pandemic baselines (5, 6). This persistent shift has renewed concerns regarding the public health implications of prolonged digital exposure, particularly with respect to rising anxiety and stress-related symptoms across diverse populations (7). As digital reliance becomes increasingly embedded in daily life, understanding its broader psychological and behavioral consequences has become an essential priority for population health research (8).

A growing body of research identifies multiple pathways linking digital behaviors to mental health outcomes (9). Extensive screen exposure, especially in evening hours, interferes with circadian rhythms, suppresses melatonin secretion, increases cognitive arousal, and reduces sleep duration and quality (10). Sleep, as a core restorative function, is central to emotional regulation and stress recovery. Its disruption may heighten vulnerability to anxiety. Social media use introduces additional mechanisms (11, 12). Passive engagement often amplifies social comparison, vigilance, and feelings of inadequacy, while algorithmically curated content environments can intensify emotional salience (13). These interrelated behavioral processes highlight the need to examine digital engagement not as isolated actions but as components of a broader behavioral ecology with implications for mental well-being. It is important to note that the majority of this evidence, and the focus of the present study, pertains to adult populations, as developmental and neurological differences may lead to distinct vulnerabilities and patterns of digital engagement among adolescents and young adults (13, 14).

The Digital Ecological Stress Model (DESM) provides a useful framework for conceptualizing how digital behaviors operate within nested ecological layers (15, 16). At the micro level, individual patterns of screen exposure and sleep directly influence emotional regulation through attentional, cognitive, and physiological processes. At the meso level, digital social environments shape experiences of comparison, evaluation, and perceived support (17). At the macro level, the post-pandemic sociotechnical environment has normalized continuous connectivity and blurred work–life boundaries, potentially magnifying digital stressors (18, 19). Despite theoretical advances (20), significant empirical gaps remain. Few studies have tested demographic moderators such as age, which may shape vulnerability (21, 22). Sleep adequacy as a buffer remains inconsistently examined. Most existing studies rely on single-year snapshots, limiting insight into post-pandemic temporal trends. In addition, variable-centered designs may obscure distinct behavioral subgroups that combine high screen exposure, insufficient sleep, and heavy social media use.

To address these gaps, the present study uses a repeated cross-sectional global dataset collected annually from 2020 to 2024 (23, 24). It examines multi-year associations between screen time, sleep duration, and social media use with anxiety, evaluates age and sleep adequacy as potential moderators, and applies person-centered clustering to identify high-risk digital behavior profiles (25). It further assesses temporal patterns to determine whether anxiety trajectories have intensified or stabilized across the five-year period. By integrating multi-year behavioral data with moderated regression and person-centered analytic techniques, this study advances the DESM framework and provides actionable public health insights. Findings may inform the development of sleep-focused behavioral interventions, digital hygiene guidelines, and age-stratified prevention strategies that support mental well-being in an increasingly connected society.

## 2 Literature review and hypotheses

### 2.1 The screen time–mental health relationship

A substantial body of meta-analytic research consistently demonstrates that increased screen exposure is associated with adverse mental health outcomes (22), particularly anxiety and depressive symptoms (26–30). These reviews frequently report a dose–response pattern, indicating that psychological risks rise as screen use exceeds recommended thresholds (28). For example, recreational use beyond approximately 2 h per day is often linked to elevated distress (12). A persistent challenge in this literature is the considerable variability in effect sizes across studies and populations (26, 29). Methodological differences, such as reliance on global screen time measures and limited attention to contextual or individual-level factors, contribute to this inconsistency (29). Although the overall association is negative, identifying the specific conditions under which this relationship becomes more pronounced remains essential (31). Based on prior evidence, we propose the following hypothesis.

*H1:* Daily screen time is positively associated with anxiety and negatively associated with happiness, independent of other factors.

### 2.2 Sleep as a Core restorative mechanism

Sleep is a critical restorative process that supports emotional regulation (32) and cognitive functioning (33, 34). Inadequate or poor-quality sleep impairs the prefrontal cortex (Gil et al., 2024). This increases susceptibility to anxiety and stress (35, 36). Digital device use, particularly in evening hours, is recognized as a potent disruptor of sleep. Exposure to blue light and pre-sleep cognitive arousal can disturb circadian rhythms (37). These processes reduce sleep quality and shorten sleep duration (38). This mechanism suggests that screen exposure influences mental health directly and indirectly by weakening the physiological systems essential for maintaining resilience (39). Sleep duration therefore represents not only an outcome of digital behavior but also a modifiable factor in the pathway linking screen exposure to psychological well-being. Accordingly, we hypothesize the following:

*H2:* Daily sleep duration is negatively associated with anxiety and positively associated with happiness, independent of other factors.

### 2.3 The dual nature of social media use

Social media represents a complex component of screen behavior with both beneficial and detrimental implications for mental health. Active engagement, such as direct communication or purposeful participation in online communities (40), can promote social support and connectedness (41, 42). These processes often function as protective factors against psychological distress. In contrast, passive consumption, such as routine scrolling or exposure to idealized self-presentations, is associated with social comparison and the fear of missing out (43, 44). These cognitive processes can fuel feelings of inadequacy and loneliness. They also impose sustained emotional demands that undermine well-being (45, 46). A nuanced understanding of digital engagement therefore requires distinguishing active, goal-directed interactions from passive content consumption. This distinction helps clarify the mechanisms through which social media influences mental health outcomes.

### 2.4 Moderation by demographic and temporal factors

The psychological impact of screen exposure is not uniform. It varies substantially across demographic and temporal contexts (47). Adolescents and young adults are widely considered more vulnerable because of ongoing neurodevelopmental maturation and heightened reliance on peer evaluation during identity formation (48). The post-pandemic period from 2020 to 2024 also represents a unique context (49). It is characterized by entrenched digital routines that may shape risk trajectories in ways not observed before the pandemic (50). Examining age and sleep adequacy as moderators is therefore essential for producing evidence-based, stratified intervention strategies (51). These considerations lead to two additional hypotheses.

*H3:* Age moderates the association between screen time and anxiety. The positive association is stronger among young adults than among older adults.

*H4:* Adequate sleep moderates the association between screen time and anxiety. The relationship is weaker among individuals who sleep seven hours or more per night.

### 2.5 Behavioral patterns and high-risk subgroups

Research relying primarily on variable-centered methods may overlook meaningful subgroups characterized by distinct combinations of digital behaviors. Person-centered analytic approaches, such as cluster analysis or latent profile analysis, can reveal behavior patterns that jointly confer risk. For example, simultaneous high screen exposure, insufficient sleep, and intensive social media use may create a specific risk phenotype associated with particularly poor mental health outcomes. Identifying these subgroups allows for more precise targeting in public health strategies and reduces reliance on broad, one-size-fits-all interventions. This approach informs our final hypothesis.

*H5:* Distinct behavioral clusters will emerge from the data. A cluster characterized by high screen time and low sleep duration will show the poorest mental health outcomes. This high-risk cluster will also exhibit increasing anxiety levels from 2020 to 2024.

## 3 Methods

### 3.1 Study design and data source

This study employed a repeated cross-sectional design, analyzing five annual waves (2020–2024) of publicly available data from the Mental Health and Lifestyle Habits Dataset (Kaggle; CC0 1.0 Public Domain license). The dataset comprises self-reported information from adults aged ≥18 years on digital behavior (daily screen time, social media use), sleep duration, psychological well-being (anxiety, perceived stress), and demographic characteristics, providing an opportunity to examine post-pandemic trends in these domains. According to the source documentation, the dataset was derived from global wellness surveys and mental health research studies and cross-verified with psychologists and wellness platforms; however, the specific timing of data collection within each year is not specified. Each wave was therefore treated as an independent cross-sectional sample, and no assumptions were made regarding potential seasonal effects, with all analyses conducted at the annual level.

Following screening for completeness and plausibility, a final analytical sample of *N* = 3,000 complete cases (approximately 600 per wave) was retained. The dataset is fully anonymized, which precludes identification or linkage of individuals across years; accordingly, analyses address population-level associations rather than individual trajectories. Geographic identifiers are not available in the anonymized file; thus, the present analyses focus on overall global associations rather than regional comparisons, with cross-national variability acknowledged as an important direction for future research. These dataset characteristics were incorporated into the analytic strategy to ensure appropriately bounded, context-sensitive interpretation of findings.

### 3.2 Measures

Anxiety was the primary outcome, measured using a single self-report item on a scale from 1 (not at all anxious) to 10 (extremely anxious), with higher scores reflecting greater anxiety. This variable was treated as continuous in all analyses. Three digital behavior variables served as primary predictors, each also treated as continuous: daily screen time, defined as self-reported hours of digital screen use per day; nightly sleep duration, defined as self-reported average hours of sleep per night; and daily social media use, defined as self-reported hours spent on social media platforms per day. Covariates included perceived stress, rated on a continuous 1–10 scale; age, derived as a continuous variable from the midpoint of the original categorical bands (18–29 years = 23.5, 30–39 years = 34.5, 40–49 years = 44.5, 50–64 years = 57.0, 65 + years = 70.0); gender, dummy-coded with male as the reference category and female and other as additional categories; and survey year, dummy-coded with 2020 as the reference.

### 3.3 Sample

Records with missing values on core variables, duplicate entries, or implausible values (screen time > 18 h/day; sleep duration < 2 or > 14 h/night) were excluded. The final analytic sample consisted of N = 3,000 complete cases, with approximately 600 respondents per year. While the survey design permitted multiple wave participation, full anonymization prevented individual tracking across years. The analytic sample comprised adults recruited via international online convenience sampling, a method commonly used in digital behavior and mental health research. Given the non-probability sampling design, the sample is not population-representative. Consequently, the analyses focus on characterizing associations among digital behaviors, sleep, and anxiety, as well as their temporal patterns, rather than estimating population prevalence. For moderation analyses, two binary moderators were created: age group (younger adults [< 40 years] vs. older adults [≥ 40 years]) and sleep adequacy (≥ 7 h/night vs. < 7 h/night, consistent with international recommendations). To minimize multicollinearity and facilitate interpretation of interaction effects, screen time, sleep duration, social media use, and perceived stress were mean-centered prior to inclusion in regression models (52).

### 3.4 Statistical analysis

All data processing and statistical analyses were performed in Python 3.11 using pandas 1.5.3, NumPy 1.24.3, statsmodels 0.14.0, and scikit-learn 1.3.0. Descriptive statistics (means, standard deviations [SD], medians, interquartile ranges) were computed overall and by survey year. Annual mean anxiety scores were visualized to examine temporal trends. Associations between digital behaviors and anxiety were examined using three hierarchical ordinary least squares (OLS) regression models with Huber–White robust standard errors. Model 1 included the three centered digital behavior predictors and all covariates. Model 2 added the interaction between centered screen time and age group. Model 3 added the interaction between centered screen time and sleep adequacy. Multicollinearity was assessed using variance inflation factors (VIF). When interaction terms were statistically significant, simple slopes were probed at each level of the moderator. All statistical tests were two-tailed, and statistical significance was set at *p* < 0.05. Behavioral profiles were identified using k-means clustering on the three z-standardized digital behavior variables (Euclidean distance). The optimal number of clusters was determined by the elbow method, average silhouette coefficient, and gap statistic; a three-cluster solution was retained (silhouette = 0.272). Cluster differences in anxiety were tested with one-way ANOVA (Welch’s correction where necessary) and Bonferroni-adjusted post-hoc tests; effect size was reported as η^2^. Temporal stability of cluster differences was examined via a robust OLS regression including cluster membership, survey year, their two-way interactions, and covariates (age, gender), with overall interaction tested by F-test.

### 3.5 Robustness checks

Clustering stability was verified through bootstrap resampling (*n* = 500) and by comparing the k-means solution with Gaussian Mixture Model–based latent profile analysis, which yielded a comparable three-profile structure. Regression results were robust to logarithmic transformation of predictors and exclusion of extreme percentile values (top and bottom 1%).

## 4 Results

### 4.1 Descriptive statistics

**Figure 1:**
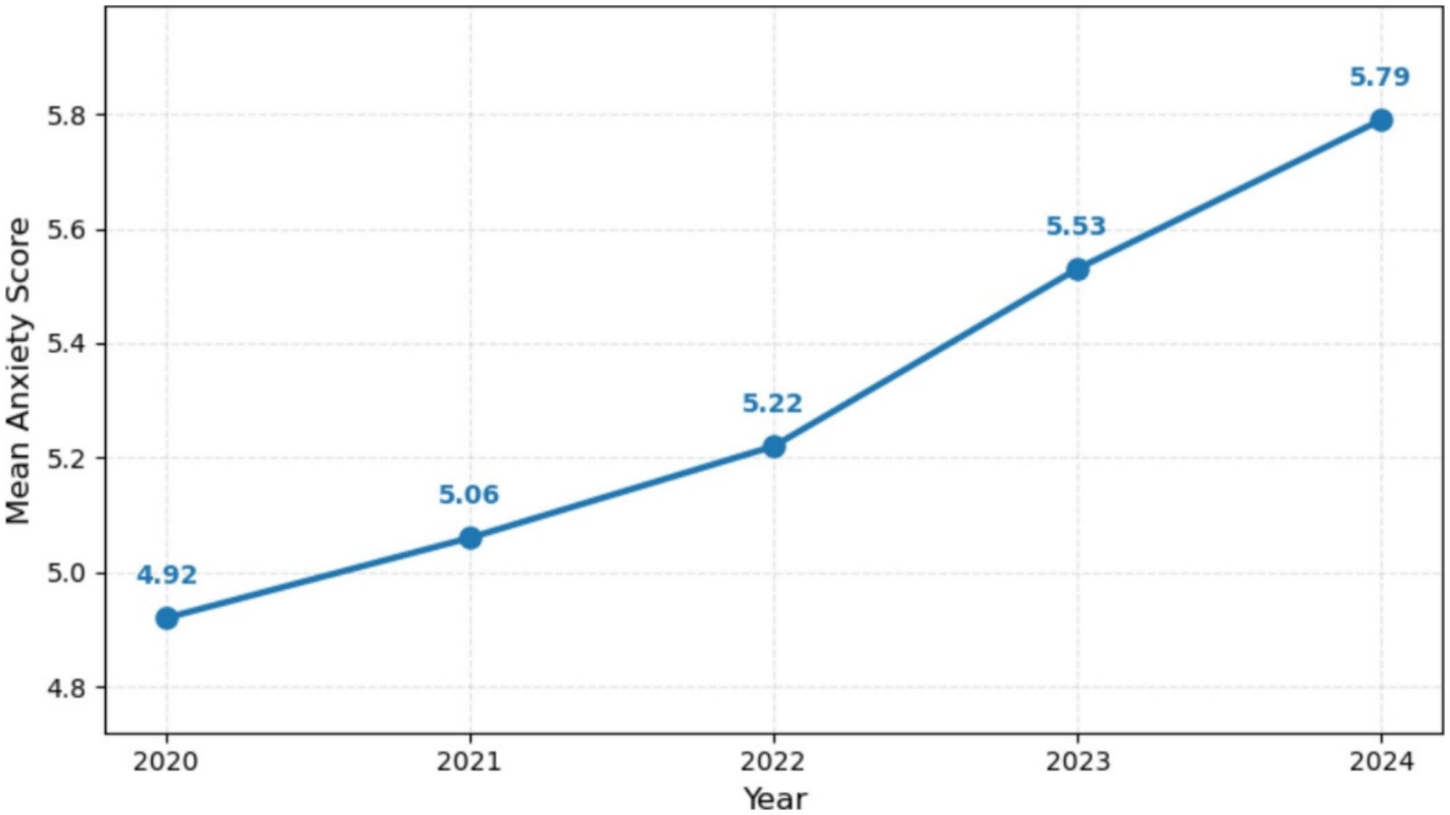
Annual mean anxiety scores (2020–2024).

Across the full sample of 3,000 participants, the anxiety score had a mean of 5.27 (SD = 1.77), with observed values ranging from 1.00 to 10.00. The median anxiety score was 5.25, with an interquartile range of 4.00–6.45. These additional indices (median and IQR) are reported alongside the mean and SD to fully characterize the distribution, which showed modest right skew. Comprehensive descriptive statistics for all primary predictors, perceived stress, and the happiness index, both for the overall sample and stratified by survey year, are presented in Table 1. Visual inspection of the annual means indicated a modest but consistent increase in anxiety levels from 2020 to 2024, as shown in Figure 1.

**Table 1:** Descriptive statistics.

Variable	2020 (*n* = 600) *M* (SD)	2021 (*n* = 600) *M* (SD)	2022 (*n* = 600) *M* (SD)	2023 (*n* = 600) *M* (SD)	2024 (*n* = 600) *M* (SD)	Overall *M* (SD)
Screen time (hours/day)	5.66 (1.79)	5.90 (1.83)	6.02 (1.87)	6.04 (1.89)	6.21 (1.92)	5.97 (1.87)
Sleep hours (nightly)	7.15 (1.44)	7.08 (1.48)	6.94 (1.52)	6.87 (1.55)	6.80 (1.61)	6.97 (1.52)
Social media Use (hours/day)	2.75 (1.10)	2.80 (1.12)	2.87 (1.14)	2.89 (1.15)	2.94 (1.18)	2.85 (1.14)
Stress level (0–10)	4.62 (2.13)	4.73 (2.19)	4.77 (2.25)	4.83 (2.27)	4.92 (2.31)	4.77 (2.23)
Anxiety Score (1–10)	4.92 (1.62)	5.06 (1.71)	5.22 (1.73)	5.53 (1.79)	5.79 (1.87)	5.27 (1.77)
Happiness index (0–10)	6.28 (1.71)	6.21 (1.74)	6.16 (1.78)	6.05 (1.82)	5.92 (1.88)	6.12 (1.79)

Screen time and social media use are reported in hours per day; sleep hours are reported in hours per night. The continuous age variable was derived from the midpoint of original categorical bands (18–29 years, 30–39 years, 40–49 years, 50–64 years, 65 + years); for example, “30–39 years” was coded as 34.5, and “65 + years” was assigned a midpoint of 70. M, mean; SD, standard deviation.

### 4.2 Hierarchical regression analyses: main effects (*N* = 3,000)

Variance inflation factors for all predictors in Model 1 ranged from 1.000 to 1.078, confirming the absence of multicollinearity. Full parameter estimates are reported in Table 2. After adjustment for gender, age, survey year, and perceived stress, all three primary digital behavior variables were significantly associated with anxiety (all *p* < 0.001).

**Table 2:** Hierarchical regression.

Predictor	*b*	*SE*	*z*	*p*	95% CI
Intercept	5.2063	0.186	27.97	<0.001	4.841, 5.571
Gender (female vs. male)	0.0368	0.057	0.65	0.518	−0.075, 0.148
Gender (other vs. male)	0.1250	0.127	0.99	0.325	−0.124, 0.374
Year 2021	0.1317	0.091	1.45	0.148	−0.047, 0.310
Year 2022	0.1638	0.089	1.85	0.065	−0.010, 0.338
Year 2023	0.3050	0.092	3.32	0.001	0.125, 0.485
Year 2024	0.3748	0.090	4.14	<0.001	0.197, 0.552
Screen time (centered)	0.3013	0.014	21.51	<0.001	0.274, 0.329
Sleep hours (centered)	−0.3608	0.018	−19.73	<0.001	−0.397, −0.325
Social media use (centered)	0.2485	0.030	8.41	<0.001	0.191, 0.306
Stress level (centered)	0.0200	0.014	1.41	0.158	−0.008, 0.048
Age (continuous)	−0.0044	0.005	−0.96	0.338	−0.013, 0.005

*b*, unstandardized regression coefficient.

Each additional hour of daily screen time was associated with a 0.3013-point increase in anxiety (95% CI [0.274, 0.329], *z* = 21.51, *p* < 0.001). Conversely, each additional hour of sleep corresponded to a 0.3608-point decrease in anxiety (95% CI [−0.397, −0.325], *z* = −19.73, *p* < 0.001). Daily social media use also showed a positive association: each extra hour predicted a 0.2485-point increase in anxiety (95% CI [0.191, 0.306], *z* = 8.41, *p* < 0.001).

Compared with the reference year 2020, anxiety scores were significantly higher in 2023 (*b* = 0.3050, SE = 0.092, *p* = 0.001) and 2024 (*b* = 0.3748, SE = 0.090, *p* < 0.001). These values represent unstandardized regression coefficients. Gender, continuous age, and perceived stress did not reach statistical significance (all *p* > 0.05). Notably, the null effect of perceived stress (*b* = 0.0200, SE = 0.014, *p* = 0.158) is considered further in the Discussion with respect to potential construct overlap, measurement limitations of the single-item scales, and shared variance already accounted for by the digital behavior variables.

### 4.3 Moderation analyses (*N* = 3,000)

**Figure 2:**
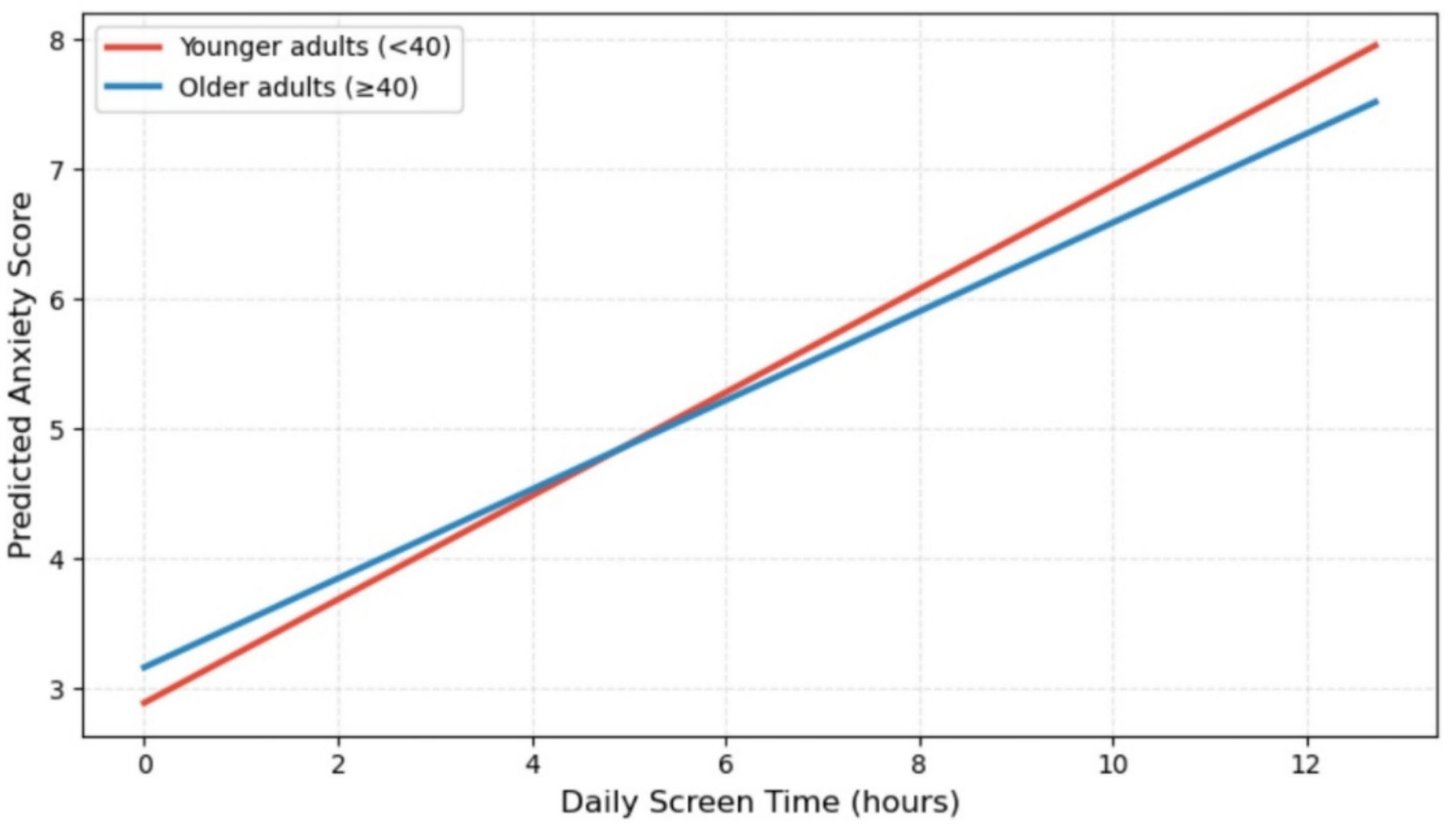
Moderation of the screen time-anxiety association by age group.

Including the interaction term between screen time and age group in Model 2 produced a statistically significant effect (*p* = 0.039). Simple-slopes analyses (Figure 2) showed that the screen time–anxiety association was significant in both age groups but somewhat stronger among younger adults (< 40 years; *b* = 0.323, *p* < 0.001) than among older adults (≥ 40 years; *b* = 0.265, *p* < 0.001). Although statistically significant, the magnitude of this interaction was modest, indicating that the two age groups do not represent sharply distinct populations.

The interaction between screen time and sleep adequacy in Model 3 was not significant (*p* = 0.121), indicating that the association between screen time and anxiety did not differ meaningfully according to whether individuals achieved seven or more hours of sleep per night. This pattern suggests that sleep may function more plausibly as a mediator rather than a moderator of the screen time–anxiety relationship—an interpretation explored further in the Discussion.

### 4.4 Behavioral clustering and profile differences (*N* = 3,000)

K-means clustering was applied to the three z-standardized digital-behavior variables, yielding a three-cluster solution with an average silhouette coefficient of 0.272. The characteristics of each profile are detailed in Table 3.

**Table 3:** Characteristics of behavioral clusters derived from *K*-means analysis.

Cluster	*n*	%	Screen time *M* (SD)	Sleep *M* (SD)	Social media *M* (SD)	Anxiety *M* (SD)	Profile label
0	1,184	39.50%	4.93 (1.57)	7.85 (1.20)	3.42 (0.50)	4.77 (1.59)	Moderate-balanced
1	1,028	34.30%	7.54 (1.54)	5.75 (1.18)	3.18 (0.66)	6.27 (1.62)	High-screen/low-sleep
2	788	26.30%	5.65 (1.80)	7.36 (1.36)	1.63 (0.48)	4.71 (1.65)	Moderate-low-social

ANOVA: *F*(2, 2,997) = 301.79, *p* < 0.001, η^2^ = 0.168 (η^2^ = partial eta-squared). Bonferroni *post hoc* tests: Anxiety in Cluster 1 > Clusters 0 & 2 (both *p* < 0.001); Clusters 0 vs. 2, *p* = 1.000.

The first cluster, designated *Moderate-Balanced* (*n* = 1,184, 39.5%), was characterized by moderate daily screen time (*M* = 4.93 h), high sleep duration (*M* = 7.85 h), and relatively high social media use (*M* = 3.42 h), with a mean anxiety score of 4.77. The second cluster, labeled *High-Screen/Low-Sleep* (*n* = 1,028, 34.3%), exhibited high screen time (*M* = 7.54 h), low sleep duration (*M* = 5.75 h), and moderate social media use (*M* = 3.18 h); this cluster reported the highest mean anxiety score of 6.27. The third cluster, identified as *Moderate-Low-Social* (*n* = 788, 26.3%), showed moderate screen time (*M* = 5.65 h), adequate sleep duration (*M* = 7.36 h), and markedly low social media use (*M* = 1.63 h), with a mean anxiety score of 4.71.

A one-way ANOVA confirmed significant differences in anxiety across the three clusters, *F*(2, 2,997) = 301.79, *p* < 0.001, with a large effect size (η^2^ = 0.168). Bonferroni-corrected post-hoc comparisons indicated that the *High-Screen/Low-Sleep* cluster reported significantly higher anxiety than both the *Moderate-Balanced* and *Moderate-Low-Social* clusters (both *p* < 0.001), whereas the latter two clusters did not differ significantly (*p* = 1.000). These findings highlight the joint role of high screen exposure and insufficient sleep in defining a higher-risk behavioral profile.

### 4.5 Temporal trends by behavioral cluster

**Figure 3:**
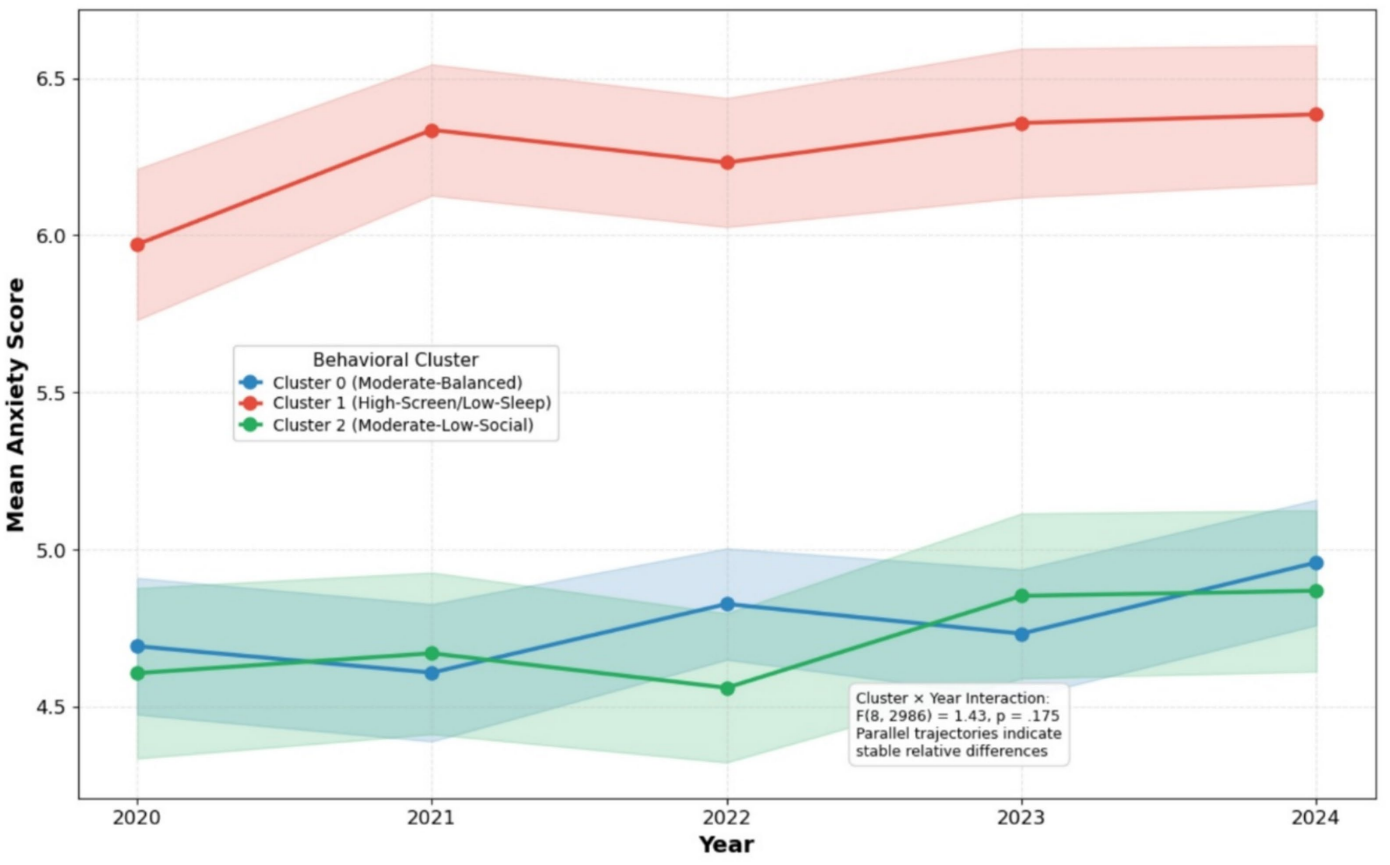
Five-year anxiety trends by behavioral cluster (2020–2024).

Finally, to examine whether the anxiety disparities between clusters changed over time, a robust OLS model tested the cluster × year interaction, adjusted for age and gender. This interaction was not statistically significant (*F*(8, 2,986) = 1.43, *p* = 0.175). As illustrated in Figure 3, the anxiety trajectories for the three clusters remained largely parallel, indicating that the between-cluster differences in anxiety were stable throughout the study period from 2020 to 2024.

## 5 Discussion

### 5.1 Principal findings and theoretical integration

This study identified consistent associations between digital behavior patterns and self-reported anxiety across the full sample. Higher daily screen time and more frequent social media use were associated with elevated anxiety levels, whereas longer sleep duration showed a robust negative association. These findings persisted after adjustment for demographic factors, survey year, and perceived stress, indicating that digital engagement patterns explain unique variance in anxiety beyond basic sociodemographic characteristics.

Importantly, these results should be interpreted as robust descriptive associations rather than evidence of causal effects. The repeated cross-sectional design allows reliable characterization of population-level patterns and group differences, but it does not permit conclusions regarding directionality or underlying mechanisms. Within this interpretive scope, the findings are consistent with theoretical frameworks proposing that digitally mediated cognitive load, attentional fragmentation, and sleep disruption jointly undermine affective regulation. For example, prolonged screen exposure may increase physiological arousal and cognitive vigilance, while insufficient sleep may impair prefrontal regulatory control and amplify emotional reactivity to digitally mediated social cues. The stronger association between screen time and anxiety observed among younger adults aligns with developmental perspectives, reflecting heightened peer sensitivity, ongoing identity formation, and deeper integration of digital platforms into daily routines. However, the modest magnitude of this interaction effect indicates graded vulnerability rather than a sharply differentiated developmental divide.

### 5.2 Examination of a non-significant moderating effect

Contrary to expectations derived from prior literature, perceived stress did not show a statistically significant independent association with anxiety in the fully adjusted regression models. This finding should not be interpreted as evidence that stress is unrelated to anxiety. Several methodological and conceptual considerations may account for this result.

First, perceived stress and anxiety share substantial conceptual overlap, particularly with respect to emotional tension, cognitive burden, and anticipatory worry. When both constructs are entered simultaneously into regression models, shared variance may attenuate the apparent unique contribution of perceived stress. Second, the use of a single-item stress measure may have limited construct coverage and reduced sensitivity to individual differences, thereby suppressing detectable effects. Third, variance associated with stress-related processes may already be partially captured by digital behavior indicators such as screen exposure and sleep duration, which are themselves closely linked to stress experiences in daily life.

Taken together, the null finding for perceived stress likely reflects measurement and model-related factors rather than the absence of a meaningful relationship. Future studies using multi-item stress scales and alternative modeling approaches may help clarify these interrelations.

### 5.3 The role of sleep: from moderation to mediation

Sleep duration emerged as a strong protective correlate of anxiety across analyses. However, sleep did not moderate the association between screen time and anxiety. Although statistically null, this finding is theoretically informative and was already anticipated in the Results section. The absence of moderation suggests that adequate sleep does not substantially buffer the immediate association between screen exposure and anxiety levels.

Conceptually, sleep may function more plausibly as an intermediary process rather than a boundary condition. Increased screen exposure, particularly during evening hours, may disrupt circadian rhythms and sleep continuity, which in turn may elevate anxiety through impaired emotional regulation and heightened physiological arousal (53). This mediational interpretation is consistent with existing sleep and affect regulation models.

Nonetheless, several methodological constraints limit this interpretation. Specifically, dichotomizing sleep duration reduces variability, a single-item measure introduces measurement error, and the repeated cross-sectional design prevents formal mediation testing or temporal sequencing. Longitudinal or experimental studies with objective sleep measures will be necessary to determine whether sleep operates as a causal pathway linking digital behavior to anxiety outcomes.

### 5.4 Behavioral profiles, effect magnitude, and stability

Beyond variable-centered associations, the behavioral clustering analysis provided additional descriptive insight by identifying interpretable profiles of co-occurring digital behaviors and sleep patterns. Individuals characterized by high screen time and low sleep duration consistently reported the highest anxiety levels, underscoring the joint relevance of these behaviors for emotional risk. Importantly, the persistence of anxiety differences between clusters across survey years suggests that these profiles reflect relatively stable lifestyle configurations rather than transient fluctuations tied to specific historical contexts.

At the same time, differences between clusters should be interpreted with appropriate caution. Although statistically robust, the separation between profiles represents broad behavioral tendencies rather than discrete or homogeneous subgroups. Notably, two clusters with markedly different levels of social media use exhibited comparable anxiety levels, indicating that social media engagement alone may be insufficient to explain emotional risk when overall screen exposure and sleep adequacy are taken into account. This pattern challenges simplified assumptions that low or moderate social media use is inherently protective and highlights the importance of considering behavioral constellations rather than isolated indicators.

### 5.5 Interpretive boundaries, limitations, and implications

Several limitations define the interpretive boundaries of the present findings and directly shape the strength of the conclusions that can be drawn. The reliance on a synthetic dataset limits ecological validity and generalizability to real-world populations. In addition, the use of single-item measures restricts construct precision and may obscure more nuanced associations, including interaction effects. Most critically, the repeated cross-sectional design precludes causal inference and constrains interpretation to stable descriptive relationships rather than developmental or mechanistic processes.

In light of these limitations, it is essential to distinguish between findings that can be considered relatively robust and those that remain provisional. The descriptive associations between digital behaviors and anxiety, as well as the identification of behavioral profiles and their temporal stability, are supported by a large repeated cross-sectional sample and represent consistent population-level patterns. In contrast, interpretations regarding moderation effects, causal directionality, and the proposed mediating role of sleep require longitudinal or experimental validation. Addressing these open questions will require future studies that incorporate longitudinal designs and objective behavioral measures, such as device usage logs and actigraphy-based sleep assessments, to establish temporal ordering and clarify underlying mechanisms.

Moreover, an important interpretive limitation stems from the exclusive focus on quantitative indicators of digital use. Time spent on screens or social media does not capture qualitative dimensions such as active versus passive engagement, content characteristics, emotional valence, or social context, all of which may differentially relate to anxiety. To advance mechanistic understanding, future research should explicitly distinguish between modes of digital engagement, contrasting active participation, including direct communication and content creation, with passive consumption, such as scrolling and exposure to algorithmically curated feeds. In addition, experimental interventions testing age-stratified digital hygiene practices and sleep-focused behavioral strategies will be essential for translating these descriptive associations into evidence-based clinical and public health recommendations.

## 6 Conclusion

High screen engagement and insufficient sleep were consistently associated with elevated anxiety, with this link being more pronounced among younger adults, while longer sleep duration demonstrated a protective relationship. Behavioral clustering further revealed that individuals with high screen time and low sleep reported the highest anxiety levels, a pattern stable across years. These findings represent robust descriptive associations that underscore the importance of a multidimensional perspective capturing co-occurring behavioral patterns and developmental context. However, given the study’s design, conclusions regarding causality, mediation pathways, and specific mechanisms remain tentative. Therefore, future longitudinal studies incorporating objective and qualitative measures are essential before these associations can reliably inform clinical or public health interventions.

## Data Availability

Publicly available datasets were analyzed in this study. This data can be found at: https://www.kaggle.com/datasets/atharvasoundankar/mental-health-and-lifestyle-habits-2019-2024.
